# Diverse Effects of Thermal Conditions on Performance of Marathon Runners

**DOI:** 10.3389/fpsyg.2020.01438

**Published:** 2020-07-03

**Authors:** Thadeu Gasparetto, Cornel Nesseler

**Affiliations:** ^1^Department of Management, National Research University Higher School of Economics, Saint Petersburg, Russia; ^2^Business School, Norwegian University of Science and Technology, Trondheim, Norway

**Keywords:** performance, marathon, heat stress, wet-bulb globe temperature, universal thermal climate index

## Abstract

Heat exposure affects human performance in many ways. Both physiological (i.e., glycogen sparing, oxygen uptake, thermoregulation) and biomechanical mechanisms (i.e., contact time, knee flexion, muscle activity) are affected, hence reducing performance. However, the exposure affects persons differently. Not all athletes necessarily experience an identical thermal condition similarly, and this point has been overlooked to date. We analyzed endurance performances of the top 1000 runners for every year during the last 12 New York City Marathons. Thermal conditions were estimated with wet-bulb globe temperature (WBGT) and universal thermal climate index (UTCI). Under identical thermal exposure, the fastest runners experienced a larger decline in performance than the slower ones. The empirical evidence offered here not only shows that thermal conditions affect runners differently, but also that some groups might consistently suffer more than others. Further research may inspect other factors that could be affected by thermal conditions, as pacing and race strategy.

## Introduction

The negative effects of heat exposure are widely known. Labor productivity is negatively impacted by heat exposure in the workplace (Dunne et al., 2013; Cheung et al., 2016; Chavaillaz et al., 2019). Previous research regarding heat exposure focuses on the effects between low- and high-risk labor (Hsiang et al., 2017), the amount of time individuals are exposed (Hancock et al., 2007), and degrees of work stress (Kjellstrom et al., 2018). A point that has been overlooked is that groups of workers within the same labor activity might experience the impacts of identical heat exposure differently (Dear, 2018).

Heat exposure and global warming have many socioeconomic impacts (Watts et al., 2018), including increased death rates (Hsiang et al., 2017; Mora et al., 2017), occupational injuries (Spector et al., 2016; Varghese et al., 2018), reduced economic production (Burke et al., 2015; Fishman et al., 2019; Martinich and Crimmins, 2019), and loss of labor productivity (Dunne et al., 2013; Zare et al., 2015). A broad literature exists discussing the negative impacts on labor productivity (Flouris et al., 2018). Physiological effects (Ladell, 1955; Hellon et al., 1956; Liu et al., 2017) are mainly associated with declines in productivity as a consequence of heat stress, but external elements such as clothing (Kampmann et al., 2012), work patterns (Yi and Chan, 2015; Takakura et al., 2017), leisure (Dyble et al., 2019), cooling systems (Tanabe et al., 2015), lifestyle (Trezza et al., 2015), and physical activity (Zander et al., 2015) can also restrain these effects.

Research has examined the impacts of global warming and heat exposure on labor productivity at different geographic levels: global (Ely et al., 2007; Dell et al., 2012; Dunne et al., 2013; Burke et al., 2015; Chavaillaz et al., 2019), national (Cheung et al., 2016; Hsiang et al., 2017; Fishman et al., 2019; Martinich and Crimmins, 2019), and regional (Barreca et al., 2016). Additionally, authors have examined these effects between gender groups (Witterseh et al., 2004), industries, income classes (Zander et al., 2015), indoor vs. outdoor conditions (Cheung et al., 2016), work risk groups (Hsiang et al., 2017), and degrees of heat stress (Kjellstrom et al., 2018). Contemporary research offers adaptation options (Day et al., 2019), limits (Sherwood and Huber, 2010), and recommendations (Watts et al., 2018) on how to cope with global warming and heat exposure.

The negative effects of heat exposure have been extensively researched in sports settings as well. Coris et al. (2004) emphasize heat and humidity as significant risk factors for human exercise. Armstrong et al. (2007) find that heat stress causing organ system dysfunction and failure is a relevant cause for athletes to withdraw from their activities. Tyler et al. (2016) find that heat adaptation is beneficial, however, they state that it depends on the duration of the training (in days). Périard et al. (2015) find, in addition to Tyler et al. (2016), that heat adaptation depends on the kind of heat (e.g., dry or humid hear) and several other factors, such as intensity or frequency. Brotherhood (2008) broadly examines the impacts of heat stress in exercise and sports. Such negative impacts are reported in such diverse sports as cycling (Tatterson et al., 2000), triathlon (Massimino, 1988; Gosling et al., 2008), baseball (Kurakake et al., 1988), and football (Mohr et al., 2012).

Being an outdoor endurance event, marathon running is a sport that researchers have examined carefully for the impacts of heat exposure. Indeed, there is robust evidence of negative effects of weather on performance of marathon runners. Numerous researchers (e.g., Trapasso and Cooper, 1989; Suping et al., 1992; Ely et al., 2007; Montain et al., 2007; Maughan, 2010; Roberts, 2010; Vihma, 2010; Miller-Rushing et al., 2012; Del Coso et al., 2013; Maffetone et al., 2017; Knechtle et al., 2018, 2019; and Nikolaidis et al., 2019b) have found that higher temperatures result in slower finishing times for both elite and amateur runners.

Although the negative impact of heat exposure is well known, it is plausible to assume that not all individuals react similarly to heat. Indeed, Dear (2018) indicates that heterogeneity among workers may result in diverse productivity under similar heat exposure. Although sport is a plausible setting in which heat exposure could have diverse effects, this important element has not been extensively studied in the previous literature. In the context of marathon running, the topic has been discussed by Ely et al. (2007), Montain et al. (2007), and Vihma (2010). They emphasize that slower runners experience larger reductions in performance than elite runners. However, their main argument is that slower runners experience more heat stress because they run for longer periods than faster runners. Our hypothesis, on the other hand, is that even though top runners run for shorter periods, they perform the task (the race) more intensely than slower runners. Hence, they could suffer more from a performance penalty than slower runners.

## Materials and Methods

We analyze runner performance in the same, constant setting (New York City Marathon) over twelve events (from 2006 to 2018 – the marathon was canceled in 2012). The New York City Marathon takes place at the first Sunday of November. All information regarding race course, rules, and results can be found at https://www.nyrr.org/tcsnycmarathon. Marathon runners are especially prone to heat stress (Roberts, 2010; Smith et al., 2016) as a consequence of the particular characteristics of a marathon, a highly physiologically demanding activity (Ely et al., 2007).

The present setting allows for an appropriate identification of impacts from thermal conditions for the following reasons: (1) goals and outputs are clear: runners seek to be as fast as they can and are ranked according to their performance; (2) similar circumstances: (2.1) all competitors run the same distance and course: 42.195 km (26.219 miles) in New York City over a period of 12 years; (2.2) all athletes are under identical thermal conditions at the same event; (2.3) no seasonal bias – every event happens on the first Sunday of November; and (2.4) no dressing bias – all runners wear similar uniforms. Previous works inspecting the impact of weather on runners analyzed different events at the same time, which undermines the identification of heat exposure effects (Ely et al., 2007; Montain et al., 2007), used air temperature and precipitation as weather conditions (Roberts, 2010; Vihma, 2010), or examined smaller samples each season (Trapasso and Cooper, 1989; Suping et al., 1992; Maffetone et al., 2017; Knechtle et al., 2018) than the current work.

### Model and Variables

We examine the impact of heat exposure on performance (*p*) by multiple linear modeling as follows:

$$p_{it}=\beta_{0}+\beta_{1}C_{jt}w_{t}+\beta_{2}C_{jt}w_{t}^{2}+\upsilon W_{it}+\varepsilon_{i}$$

where *p* is performance, measured by the finishing time in seconds; *i* is an individual runner; *t* is a year; *C* is a matrix of clusters *j* = 1, 2, 3, or 4; *w* is thermal condition, measured by WBGT or UTCI, both in Celsius scale (°C), at the beginning of each marathon; ***W*** is a vector of control variables. The control variables are age, gender, nationality, number of previous participations, the defined clusters, and start corral dummy (since 2008). An alternative model inspecting a quadratic age effect (cf., Lara et al., 2014; Nikolaidis et al., 2019a) is also carried out but both terms – first and second order – shows insignificant effect and does not influence the results. Start corral was established in the New York Marathon in 2008 with the aim to offer safer conditions for runners by an ordered and smooth flow. It is currently the standard start approach in most major marathon events worldwide. The variables of interest are two interaction terms in each equation: interactions between a heat exposure index (WBGT or UTCI) and its squared term and the defined clusters. It aims to capture the nonlinear impact that heat exposure might have on productivity, as observed by Burke et al. (2015). We use temperature in Celsius degrees throughout the paper. Fahrenheit degrees are related as follows: F =°C ^∗^ 1.8000+ 32.00.

Thermal condition is calculated by the WBGT and the UTCI at the start time of each marathon. WBGT is the most popular measure of heat exposure (Epstein and Moran, 2006; Kjellstrom et al., 2009; Lemke and Kjellstrom, 2012) and is currently an ISO standard (ISO, 2017). UTCI is a more recent index that affords a slightly different measure of thermal perception (Zare et al., 2018). Brocherie and Millet (2015) discuss some potential limitations regarding WBGT by measuring the severity of the thermal conditions on sport practice. They indicate that UTCI is more appropriate for sport heat stress modeling. However, most of the recent literature still uses WBGT as a bioclimatic index. Therefore, we decided to include both. Both indexes incorporate temperature, solar radiation, wind speed, and humidity, but differ in their calculations. Specifically, WBGT is calculated as follows:

$$WBGT=0.7*T_{w}+0.2*T_{b}+0.1*T_{d}$$

where *T*_*w*_ is wet-bulb temperature; *T*_*b*_ is black globe temperature; and *T*_*d*_ ambient temperature (Epstein and Moran, 2006). UCTI is calculated as follows:

$$UTCI=f\left(T_{a};T_{mrt};va;RH\right)=T_{a}+Offset\left(T_{a};T_{mrt};va;RH\right)$$

where *T*_*a*_ is air temperature; *T*_*mrt*_ is air mean radiant temperature; *va* is wind speed; and *RH* is relative humidity (Błażejczyk et al., 2013). Air temperature, solar radiation, wind speed, and humidity are the inputs in our calculations. Brocherie and Millet (2015) suggest these basic weather elements for WBGT calculation. Both indexes were automatically calculated using the heat stress calculator available at http://www.climatechip.org.

**FIGURE 1:**
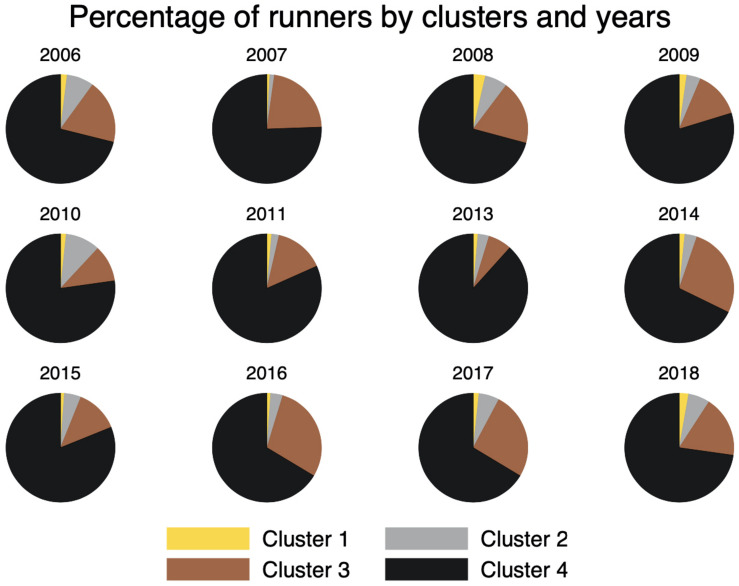
Percentage of runners by clusters and years.

The data comprises the top-1000 overall runners for each event. This sample represents about 2% of total participants for each year. Of these, 89.70% completed the race in less than 3 h – the slowest runner in the sample finished in 3:04:43. Previous work habitually uses subjective criteria to separate runners into groups (e.g., top-3, top-10, top-50, and top-100). Nevertheless, such an arbitrary definition is bound to lead to misspecifications. For instance, the 11th place in a year might be more similar to the runners of the top-10 *group* than compared to the top-50 group. Therefore, to appropriately examine whether groups experience thermal conditions differently, runners were clustered by their performance on each event. The hierarchical clustering through the average linkage method was used to establish the clusters. This method is chosen because of the hierarchical, top-down distribution of runners according to finishing times. The method clusters the data by considering the distance between two clusters as equal to the average distance from any members of both clusters (Ward, 1963; Rokach and Maimon, 2005). There are 222 observations (1.85%) in Cluster 1; 600 observations (5.00%) in Cluster 2; 2192 observations (18.27%) in Cluster 3; and 8985 observations (74.88%) in Cluster 4. Figure 1 shows the percentage of runners for every cluster for every year. Reasons for changes could be, e.g., the (non-)participation of some runners in specific years, the increase (decrease) in their performances, injuries prior or during the events, or several other external elements that we cannot capture with our data.

## Results and Discussion

**FIGURE 2:**
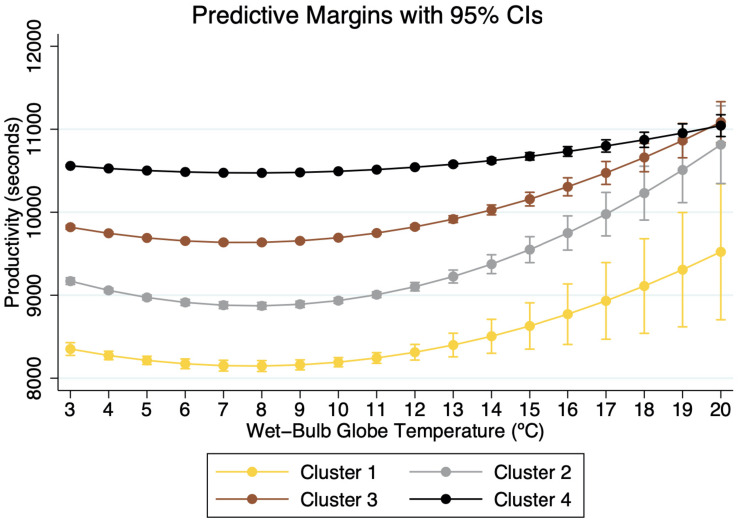
Marginal Impact of WBGT on performance by clusters.

**FIGURE 3:**
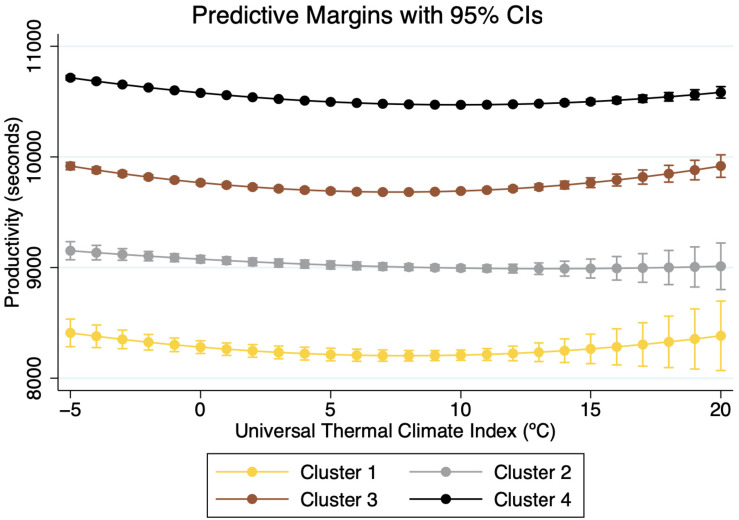
Marginal Impact of UTCI on performance by clusters.

The output of the regressions is given in Table 1. All control variables show the expected coefficients. The results also confirm the nonlinear effects of thermal conditions on performance (Burke et al., 2015). In line with previous findings, higher temperatures reduced the performance of runners. However, the results show that each cluster reacts to identical thermal conditions differently. Figures 2, 3 show the predictive marginal effects of WBGT (°C) and UCTI (°C) by each cluster. We choose the range of values of temperature (*x*-axis) from the minimum valid value of WBGT and UTCI in our sample (+3°C and −5°C, respectively) up to the maximum “Do not start” (DNS) temperature for a marathon (+20°C WBGT) (Roberts, 2010).

**TABLE 1:** Effects of WBGT and UTCI on performance by clusters of runners.

Variables	Dependent variable: Performance	
	(*w* = WBGT)	(*w* = UTCI)
Cluster 1*Heat Index	−141.7***	−19.58***
	(40.83)	(7.60)
Cluster 2*Heat Index	−202.9***	−12.89**
	(23.54)	(5.24)
Cluster 3*Heat Index	−138.3***	−22.56***
	(12.63)	(2.06)
Cluster 4*Heat Index	−58.75***	−21.82***
	(6.55)	(1.22)
Cluster 1*Heat Index^2^	9.16***	1.23*
	(2.74)	(0.73)
Cluster 2*Heat Index^2^	13.03***	0.48
	(1.58)	(0.51)
Cluster 3*Heat Index^2^	9.25***	1.50***
	(0.84)	(0.22)
Cluster 4*Heat Index^2^	3.79***	1.10***
	(0.44)	(0.12)
Cluster 2	967.1***	794.7***
	(148.3)	(33.13)
Cluster 3	1,457***	1,486***
	(136.0)	(30.74)
Cluster 4	2,007***	2,298***
	(131.4)	(29.73)
Previous Participations	−23.47***	−23.36***
	(1.66)	(1.63)
Age	5.27***	5.02***
	(0.35)	(0.34)
Gender	51.61***	47.17***
	(10.16)	(10.01)
Start Corral	−77.97***	−90.81***
	(7.15)	(6.77)
Constant	8,893***	8,539***
	(294.6)	(261.8)
Nationality	Yes	Yes
Observations	11,999	11,999
Adjusted R-squared	0.80	0.81

Standard errors in parentheses. ***p < 0.01, **p < 0.05, *p < 0.1.

The general result is that higher temperatures reduce performance in marathons, corroborating previous findings (Ely et al., 2007; Roberts, 2010; El Helou et al., 2012; Miller-Rushing et al., 2012; Del Coso et al., 2013; Cheung et al., 2016; Maffetone et al., 2017; Knechtle et al., 2018). The tipping point of productivity (about +8°C WBGT) is similar to what has been found before. Montain et al. (2007) showed a progressive decrease in performance in temperatures above 5–10°C WBGT, while Knechtle et al. (2018) observed reduced performances of runners under air temperatures higher than +8°C.

However, the current work refutes earlier research that suggests that faster runners suffer less than slower runners (Ely et al., 2007, Montain et al., 2007; Vihma, 2010). In earlier research, the argument was made that slower runners are exposed to heat stress for longer periods and hence suffer more. However, we assume that when analyzing all participants in a marathon, the slower runners (mainly amateurs) tend to have lower performances under higher temperatures not only because of the time exposed, but also due to their inferior level of training and weaker physiological adaptations and metabolic rates than elite runners. Therefore, a comparison among a relatively more homogeneous group of runners, as performed in the current work, is needed to understand whether thermal conditions impact them differently as well.

Our results indicate that top runners suffer higher decreases in performances than slower ones. As shown in Figures 2, 3, when comparing finishing times under optimal (+8°C WBGT) and DNT temperatures (+20°C WBGT), the present results indicate an average decrease of about 1,376 s (16.89%), 1,942 s (21.89%), 1,149 s (15.04%), and 569 s (5.44%) for groups 1, 2, 3, and 4, respectively. Faster runners perform the same task at a higher intensity than slower ones, and therefore thermal conditions affect their performance more than the slower groups. Del Coso et al. (2013) previously suggested that runners with higher levels of fatigue could not sustain their habitual pace, and accordingly their performance deteriorates. Therefore, although less skilled runners spend longer periods under heat exposure, their relatively less intense activity explains, to some extent, the reduced negative impact from higher temperatures when compared to top runners.

Psychological elements would also be associated with such performance penalties for faster runners. World-class and elite runners – the fastest in a major marathon – are in a highly competitive situation: pressure to win, earning places in other major events, or achieving certain results because of financial incentives from sponsors. These performance pressures in sports usually result in injuries (Strauss and Curry, 1983) and in some cases may drive top athletes to behave unethically (Volkwein, 1995; Barroso et al., 2008). Donahue et al. (2006) shows that intrinsic and extrinsic motivations are appropriate to prevent elite athletes from unethical behaviors, but they do not eliminate the pressure for good results. Therefore, top-level runners would try to surpass their physiological limits to cope with the negative effects of heat stress, and hence may suffer a bigger performance penalty than slower runners under identical conditions. Unfortunately, our data does not allow us to investigate this matter further, but additional studies are encouraged that focus on these types of questions.

Further research could also exploit the limitations of this paper. Doherty et al. (2020) assess the effect of many training elements on marathon performance but, because of a lack of data availability, they were not included in the present research. Indeed, certain training patterns might help coping with negative effects of heat stress. Pacing and other race strategies are also important elements in marathoners’ performance, but were not available for the current research. Further research could examine how heat exposure influences pacing for different groups of athletes as well as analyze optimal strategies that handle certain environmental conditions during marathons. Studies focusing on thermal impacts on gender and age groups are also encouraged. Finally, the variation of thermal conditions during a marathon is an interesting factor that certainly impacts race performance and that should be examined as well. Understanding these elements is essential to maximize sports performance as well as assure the safety of runners.

Several practical implications from this research are already reported in the literature. However, our output is very relevant supporting previous recommendations. Marathons should happen under temperatures around +8°C WBGT, assuring higher levels of performance and safety for all participants. Additionally, organizers should avoid competitions with thermal conditions over +20°C WBGT. The marginal effects presented here can help runners to adjust their training sessions considering the thermal conditions. We may assume that the negative impacts might be similar for both training and competition and, therefore, runners should be aware of these performance penalties not only on the race day, but also during their whole preparation. Finally, the evidence that faster runners suffer from a greater performance penalty than slower ones under identical thermal conditions suggests an opportunity for performance’ optimization for top-runners. Researchers and athletes together have to search for ways to maximize performance under such conditions.

The results here are important for future research regarding the impact of thermal conditions on performance. We demonstrate that marathon runners suffer from thermal conditions in different ways: under identical thermal conditions, top runners experience a substantially greater performance decrement than slower runners. This finding, which contradicts previous research, is the key contribution of the present research. Marathon is a genuine competitive sport, which makes these results relevant for other sport settings (e.g., triathlon, cross-country skiing, cycling) (Orr, 2020). However, the results are not exclusively valid for the sport sphere since heterogeneous performance levels can be found in numerous settings where individual-, group-, and firm-variation is regularly observed. For instance, further research is encouraged that investigates the physiological responses of different groups of professional workers – both athletes and non-athletes – based on their skill level. Researchers should be aware of these elements and examine as precisely as possible each specific case prior to performing estimations for future scenarios. Understanding why groups experience heat stress differently under identical circumstances is crucial for relevant scientific and policy recommendations.

## Data Availability Statement

All data used in this research are publicly available and open access. Productivity data are available at http://www.marathonguide.com and weather data are available at https://maps.nrel.gov/nsrdb-viewer/.

## Author Contributions

All authors listed have made a substantial, direct and intellectual contribution to the work, and approved it for publication.

## Conflict of Interest

The authors declare that the research was conducted in the absence of any commercial or financial relationships that could be construed as a potential conflict of interest.
